# Skeletonized internal thoracic artery harvesting: a low thermal damage electrosurgical device provides improved endothelial layer and tendency to better integrity of the vessel wall compared to conventional electrosurgery

**DOI:** 10.1186/s13019-018-0797-3

**Published:** 2018-10-11

**Authors:** Alicja Zientara, Paul Komminoth, Burkhardt Seifert, Dragan Odavic, Omer Dzemali, Achim Häussler, Michele Genoni

**Affiliations:** 1 Department of Cardiac Surgery, Triemli Hospital, Birmensdorferstrasse 497, 8063 Zurich, Switzerland; 2 Department of Pathology, Triemli Hospital, Birmensdorferstrasse 497, 8063 Zurich, Switzerland; 3 Department of Cardiac Surgery, University Hospital Zurich, Rämistrasse 100, 8001 Zurich, Switzerland

**Keywords:** Internal thoracic artery harvesting, Arterial graft patency, Electrosurgery, Arterial graft preparation

## Abstract

**Background:**

Electrosurgery is fundamental to the precise, fast and bloodless preparation of internal thoracic artery grafts in cardiac surgery. The PEAK PlasmaBlade is a monopolar electrosurgical device that uses pulsed radiofrequency energy to generate a plasma-mediated discharge along an insulated electrode, creating a cutting edge while the blade stays near body temperature. The aim of this study is to compare the histological samples, cardiac computed-tomography of graft patency, and clinical outcomes of patients after off-pump coronary artery bypass grafting with preparation of the internal thoracic arteries by a conventional electrosurgical device and the PlasmaBlade.

**Methods:**

In twenty subjects one internal thoracic artery was prepared with PlasmaBlade and the other artery with a conventional electrosurgical device. Histological samples were evaluated for three factors for potential graft failure: endothelial damage, integrity of the vessel wall and adventitial hemorrhage. Five samples per artery were evaluated by a novel scoring method based on the exposed circumference of the histological sample (“0”: 0%, “1”: 1–25%, “2”: 26–50%, “3”: 51–75%, “4”: ≥76% of the circumference). The Wilcoxon signed ranks test for mean scores within subjects was performed. Six-month-follow up by cardiac computed tomography for evaluation of graft patency was completed in 16 patients.

**Results:**

Histological results demonstrated significantly less endothelial damage after PlasmaBlade (83% vs 60%, absolute: 75/90 vs. 53/89 samples with score “0–1”, *p* = 0.04). PlasmaBlade samples demonstrated a tendency to better wall integrity (72% vs. 54%, absolute: 64/89 vs. 47/87 samples with score “0–1”, *p* = 0.32). There were no differences in endothelial bleeding (PlasmaBlade 46% vs. electrosurgery 53%, absolute: 41/88 vs. 48/90 samples with score “0–1”, *p* = 0.63). Computed tomography confirmed non-inferiority of the PlasmaBlade to conventional electrosurgery with a patency rate of 94%.

**Conclusion:**

Histologically, internal thoracic arteries harvested with PlasmaBlade demonstrate a more intact endothelial layer and a tendency to better wall integrity. Computed tomography of graft patency speaks for non-inferiority to conventional electrosurgery. PlasmaBlade may be preferable to conventional electrosurgery, if further follow-up confirms patency of internal thoracic arteries.

**Trial registration:**

NCT03510026, registered 4th April 2018 (retrospectively registered).

## Background

Electrosurgery is fundamental to the precise, fast and bloodless preparation of internal thoracic artery grafts in cardiac surgery. The fundamental performance of electrosurgical dissection is created by using a continuous radiofrequency energy waveform, which thermally ablates soft tissue, leaving a collateral damage zone of 100–400 μm [1]. The basic mechanism of tissue ablation and dissection in electrosurgery involves Joule heating of the conductive tissue by electric current, that leads to vaporization and ionization of the water content in the tissue adjacent to the electrode, and ultimately to vapor expansion and tissue fragmentation [1, 2]. Tissue heated below the vaporization threshold remains in place, but can undergo thermal denaturation determined by the temperature levels and duration of the hyperthermia. Thus, to confine the collateral damage zone in tissue, both of these factors should be minimized.

In contrast to continuous radiofrequency energy, pulsed electric waveforms with burst durations ranging from 10 to 100 μsec applied via an insulated planar electrode with 12 μm wide exposed edge produces a plasma-mediated, precise dissection of tissues with a lower collateral damage zone ranging from 2 to 10 μm. The greatly reduced zone of thermal damage, compared to conventional electrosurgical devices, may provide faster healing and less scarring [3].

**Fig. 1:**
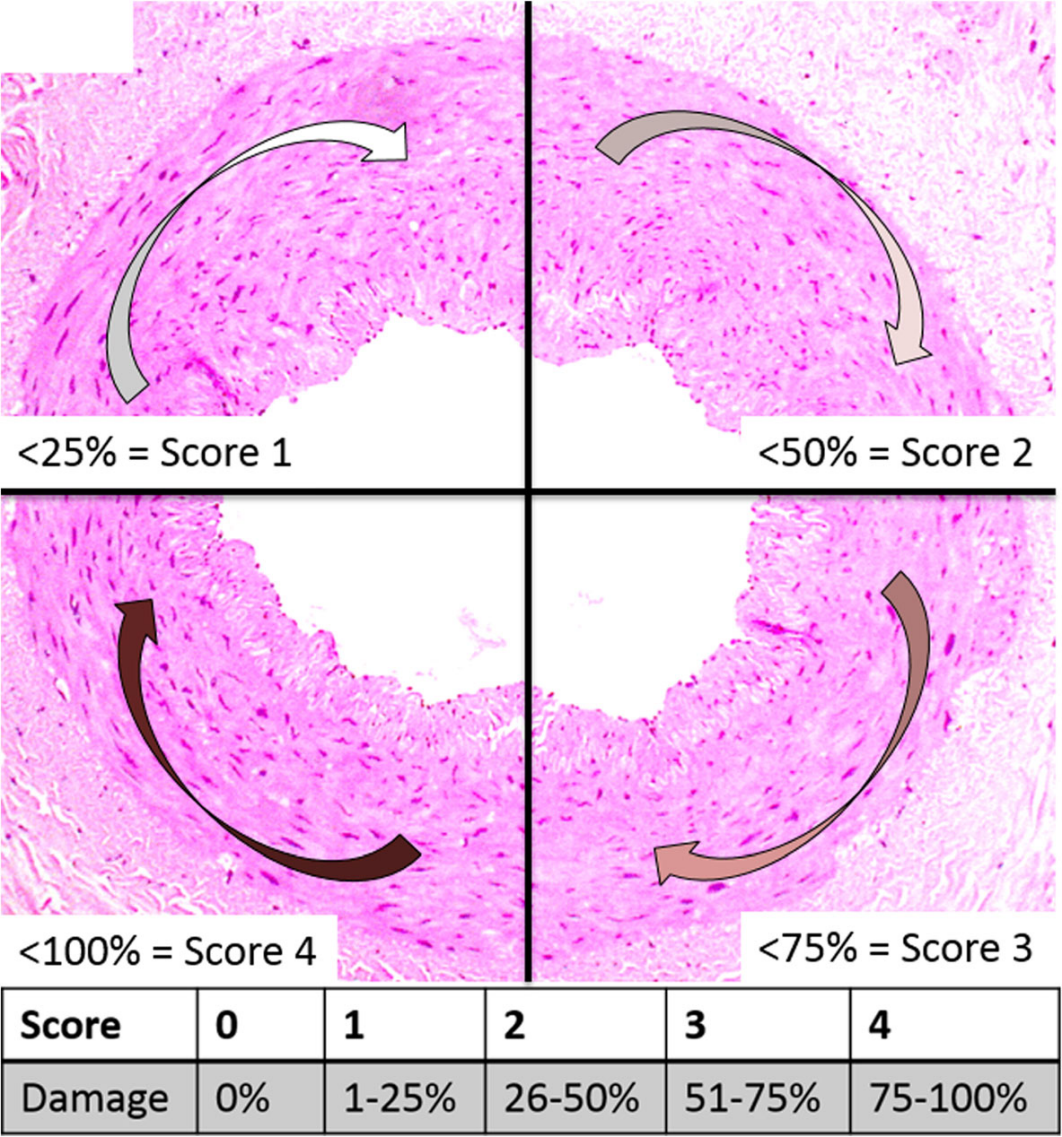
Circumference score

The PEAK PlasmaBlade (Medtronic Advanced Energy, Portsmouth, NH USA) (FDA 510(k), CE-No. 540861, Model Number PS200–040) is an electrosurgical device that uses pulsed radiofrequency energy to generate a plasma-mediated discharge along the exposed rim of an insulated blade, creating an effective, precise cutting edge while the blade stays near body temperature (Fig. 1, Table 1). Plasma is an electrically conductive cloud created when the energy contacts tissue. This conductive cloud or “plasma” allows the radiofrequency energy to cross at much lower overall power levels. This use of less energy via plasma results in lower operating temperatures and less thermal damage [4]. This technology has been shown to effectively dissect ophthalmologic and cutaneous tissues as precisely as a scalpel with the hemostatic control of conventional electrosurgery in clinical and experimental settings [5–8].

**Table 1:** Report 71-10-2475, PlasmaBlade Operating Temperature - Summary

Conventional electrosurgery temperature					
Power (Watt)	10 W	20 W	30 W	40 W	50 W
Cut mode (C°)	157.7	198.8	237.7	245.3	320.3
Coag mode (C°)	151.3	206.4	203.7	196.8	293.6
PEAK PlasmaBlade temperature					
Setting	1	2	3	4	5
Cut mode (C°)	26.2	43.5	75.9	81.8	92.7
Coag mode (C°)	2.2	91.7	99.2	115.7	111.9

Medtronic Advanced Energy, Portsmouth, NH; Mean temperature during 5 s incision of chicken breast, measured by infrared camera

Concentrating on bypass grafts, the thoracic internal arteries (ITAs) demonstrate our most valuable conduit for revascularization of the coronary arteries. Compared to pedicled arteries, skeletonized ITAs have demonstrated a tendency to better long term patency [8–11]. Additionally, skeletonized conduits are useful in expanding the number of anastomoses per patient and reducing the incidence of sternal complications [11–13].

The use of a dissection device that provides precise preparation, including optimal bleeding control without overly damaging the surrounding tissue, might be an optimizing factor for the protection of these valuable bypass grafts. The aim of this study was to compare the histological assessment, cardiac computed-tomography and clinical outcomes of patients following off-pump coronary artery bypass grafting with preparation of the ITAs by conventional electrosurgery and the PlasmaBlade.

## Methods

The study was approved by the Cantonal Ethics Committee of Zurich with the registration number KEK-ZH-Nr. 2013–0017. The period for clinical data collection was established from August 2013 till August 2014.

### Study design

This prospective, experimental study included twenty subjects that underwent coronary artery bypass grafting (off-pump) with both internal thoracic arteries. In each patient one artery was prepared with the conventional electrosurgical device (EC) (Covidien, Valleylab Reliant Pencil™, FDA 510(k), CE-No. 0086, Model Number VL2600DB) and the other with the PlasmaBlade (PB). All arteries were harvested by the same surgeon. Instrument settings for the PlasmaBlade were “Coagulation” (Setting: 5) and “Cut” (Setting: 1). These settings correspond to different electrode temperatures and provide a reduced operating temperature compared to traditional electrosurgery (Table 1). Settings for the PB were chosen by the surgeon as the most practicable settings for preparation. The standard instrument setting for ITA preparation by conventional electrosurgery was “Coagulation” (Setting: 20 W). Before sternotomy, we did a random selection, which of the ITAs is going to be prepared with the PlasmaBlade or the standard electrocautery device. After inclusion of all patients, we had a balanced distribution of 10 right ITAs and 10 left ITAs harvested by each device, resulting in cumulatively 40 grafts. After complete preparation of the ITA down to its’ epigastric bifurcation, the arterial sample of 5 cm has been extracted from the distal part of the ITA before the branching into the epigastric bifurcation. The ITA samples were immersed in 10% neutral buffered formaldehyde and embedded in paraffin for blinded, permanent histological analysis by a pathologist. Additionally, time to complete artery harvest was recorded for both devices.

### Study endpoints and null hypothesis

The primary endpoints of the study included assessment of wall integrity, presence of adventitial bleeding, and continuity of the endothelial layer of the internal thoracic arteries by permanent histology. The secondary endpoint was defined as patency of the internal thoracic artery bypass graft as assessed by cardiac computed-tomography at 6 months following surgery. The null hypothesis was that the primary and secondary endpoints would be equivalent for internal thoracic artery bypass grafts harvested by the PB or the conventional electrosurgical device.

### Histological investigations

**Fig. 2:**
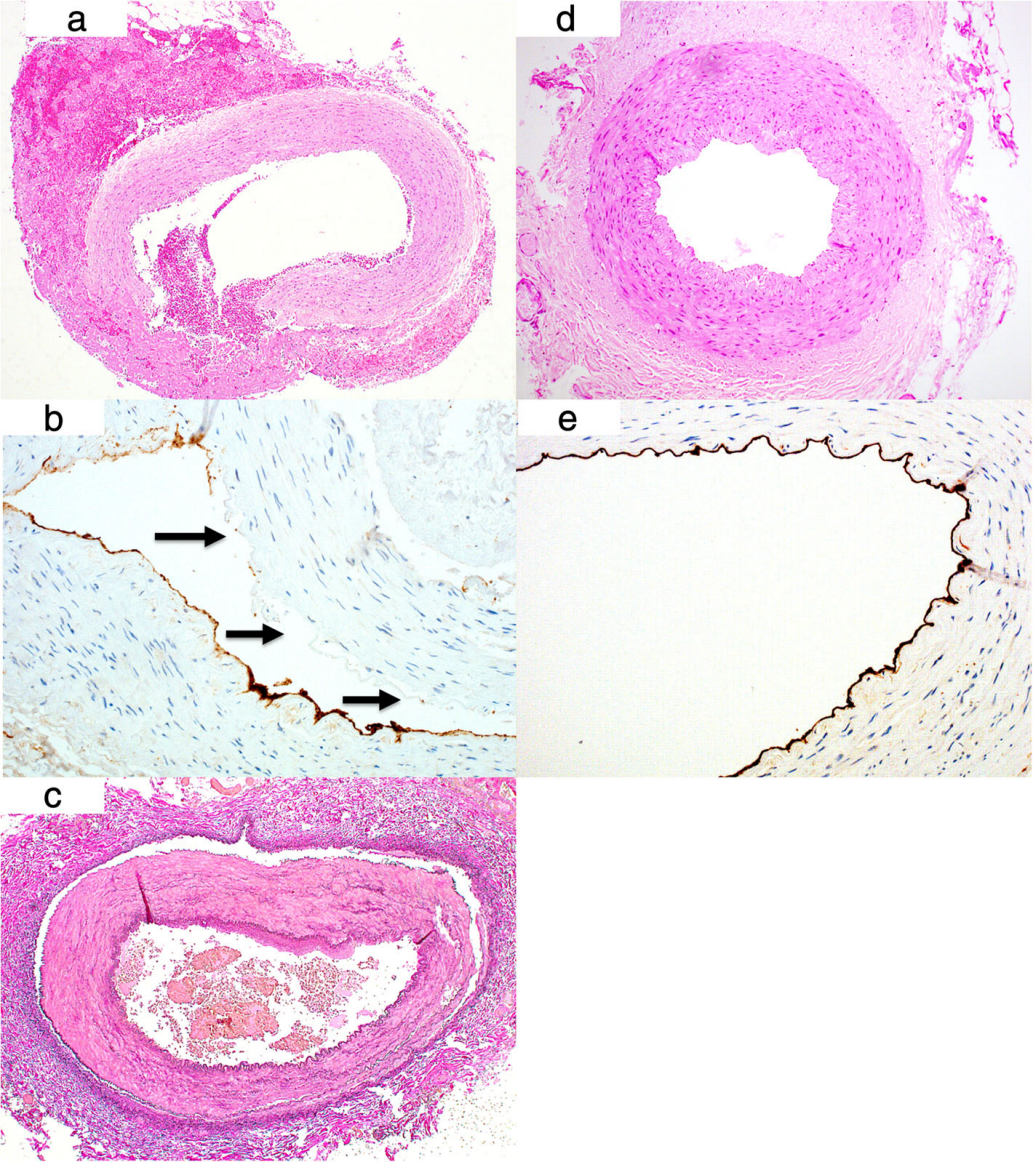
**a** Hemorrhage with intraluminal breakthrough in an ITA prepared by conventional EC; extension of the bleeding of over 50% of the circumference = score “3”, (H.E.-staining, magnification × 50). **b** Endothelial damage in an ITA prepared by the conventional EC; arrows show intraluminal spots without endothelium (score “3”), (CD31-immunostaining, magnification × 100). **c** Wall dehiscence; separation of the vessel layers demonstrating an interruption in wall integrity in the media and adventitia over 75% of the circumference (score “4”); prepared by conventional EC (H.E.-staining, magnification × 50). **d** Preserved vessel wall; perfect integrity without adventitial bleeding (score “0”); prepared by PB (H.E.-staining, magnification × 50). **e** Preserved endothelial layer covering the whole circumference (score “0”); prepared by PB, (CD31-immunostaining, magnification × 100)

Three routine stains (haematoxylin and eosin (H&E), elastica van gieson (EvG), Alcian blue-PAS (periodic acid-Schiff)) and the immunohistochemical stain CD31 were used for light microscopy (Imager A1 Zeiss microscope, AxioCam MRC5) (Fig. 2a-e). Five samples of 5 μm sections were cut and analyzed for each ITA. A blinded evaluation of adventitial bleeding, vessel wall dehiscence and endothelial integrity was performed by a single pathologist using 100× and 50× magnification. Only samples with uninterrupted circumference were chosen for evaluation.

### Circumference score and statistical analysis

**Fig. 3:**
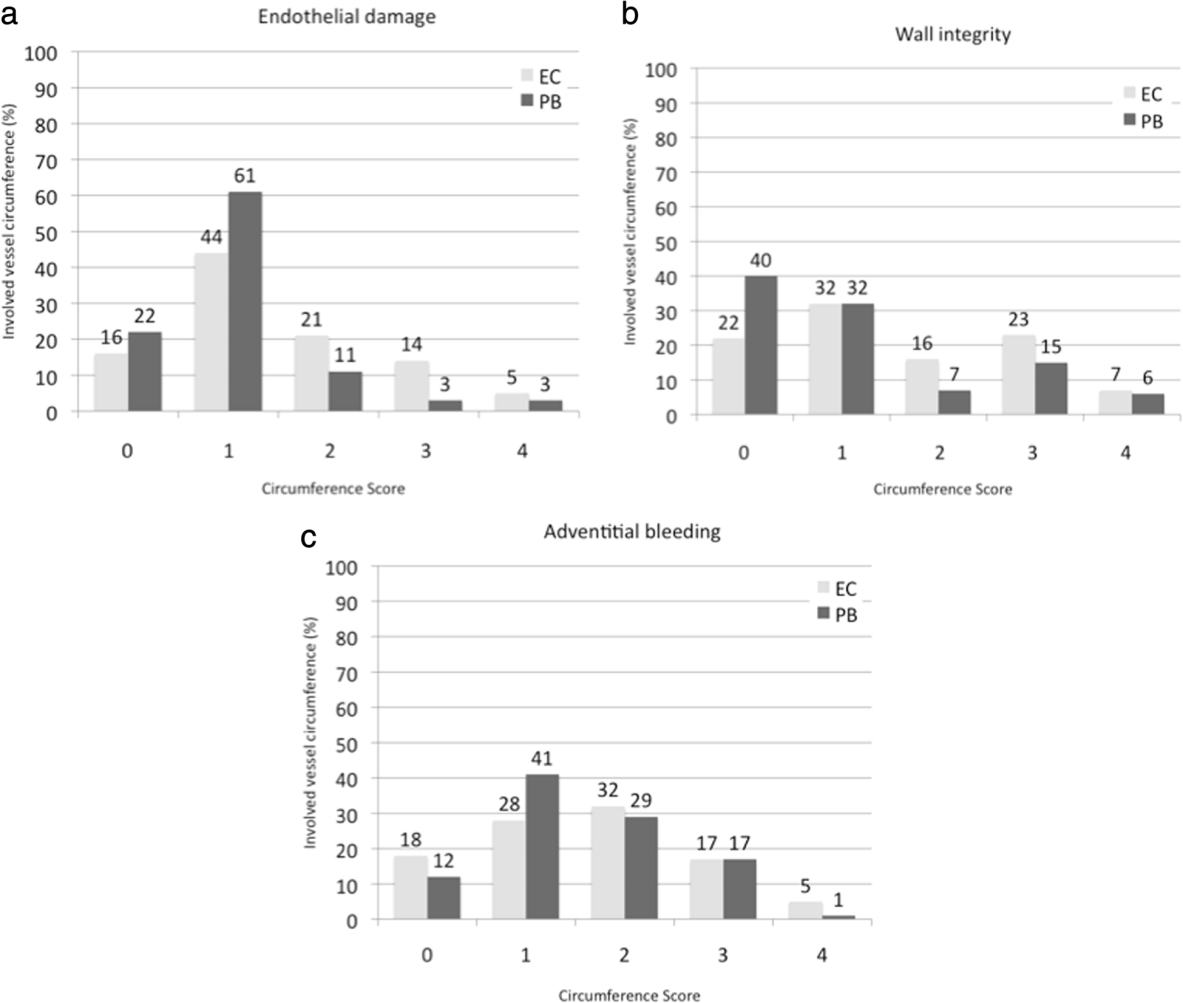
**a** Endothelial damage; significant reduction in endothelial damage in the PB group. **b** Wall integrity; tendency to better wall integrity in the PB group. **c** Adventitial bleeding; no differences between both devices

A scoring system based on the division of the vessel circumference into sections was created (Fig. 3). We distinguished between a vessel with complete integrity of the entire circumference (Score “0” = 0% of circumference is damaged), and vessels with affected sections (“1” = 1–25% of circumference, “2” = 26–50%, “3” = 51–75%, “4” = over 75% of circumference). The average of five samples from one ITA constituted the final score (minimum “0”; maximum “4”) for wall dehiscence, adventitial bleeding and endothelial damage (Fig. 2a-c). Mean scores within patients were compared between devices using the Wilcoxon signed ranks test.

### Cardiac computed tomography

Six months after the initial operation, a cardiac computed tomography (GE Healthcare, GE Discovery 750 HD) scan was performed to detect graft failure and evaluate patency. Patients with newly diagnosed renal failure or refusal of the investigation were excluded from the computed tomography (CT). A single radiologist, blinded to the test instrument, evaluated all images.

## Results

### Histological results

#### Less endothelial damage

The histological results demonstrated a statistically significantly reduction in endothelial damage in samples harvested with the PB (83% vs. 60%, absolute: 75/90 versus 53/89 samples with a score of “0–1”, *p* = 0.04, Fig. 3a). The majority of the PB endothelial damage samples show 0% to less than 25% involvement, of the circumference of the graft. Comparatively, 21% of the conventional electrosurgery samples demonstrated endothelial damage between 25 and 50% of the circumference. Samples with a score over “3”, indicating severe endothelial damage of more than 50% of the circumference, comprise 19% of the EC group vs. merely 6% of the PB group.

#### Tendency to better wall integrity

ITA samples harvested with the PB demonstrated a tendency towards better wall integrity (72% vs. 54%, absolute: 64/89 vs. 47/87 samples with a score of “0–1”), which was not significant in the Wilcoxon signed ranks test (*p* = 0.32, Fig. 3b). However, approximately 40% of the PB samples demonstrated perfect wall integrity with a score of “0” compared to only 22% in the conventional electrosurgery group. In both groups, the percentage of samples with a score of “1” was similar with 32%. Cumulatively, samples with a score of “2” or higher comprised approximately 46% of the conventional electrosurgery samples and only 28% of the PB samples.

#### Adventitial bleeding

There was no difference in the percentage of samples with observed endothelial bleeding with a similar distribution of the samples to the circumference score between the two test instruments (*p* = 0.63, PB 46% vs. EC 53%, absolute: 41/88 vs. 48/90 samples with a score of “0–1”) (Fig. 3c).

#### Cardiac computed tomography after 6 months

Sixteen of twenty possible follow-up CTs were performed. One patient suffered from postoperative renal failure and could not undergo the investigation. Two patients refused the CT and one patient died at the first postoperative day in the intensive care unit with progressive multiorgan failure as a result of a fulminant vasoplegic shock and emergent installation of extracorporal circulatory membrane support most likely because of an anaphylactic reaction. The resultant fourteen CTs confirmed a good patency of both ITAs. One CT showed an occlusion of the right ITA, which was prepared by PB, that was anastomosed to the right coronary artery. On another CT, neither ITA could be localized. In summery, in 14 of 15 evaluable CTs the PB ITAs showed a good patency, which means a patency rate of 94%.

#### Preparation time

Preparation of the ITA by PB took significantly longer with a mean of 26.3 min (standard deviation ±5.2 min) in comparison to the EC with 21.2 min (standard deviation ±4.4 min) (*p* = 0.001).

## Discussion

Preparation of tissue using different devices started with the aim to reduce complications like bleeding, poor wound healing and infection. Multiple electrosurgical devices have been investigated for skin dissection in this manner, and have since found their way to more complex procedures in laparoscopic surgery or graft harvesting [14]. The ITA has traditionally been harvested with a monopolar electrosurgical device using a pedicled technique. With the trend to skeletonizing the ITA in light recent investigations demonstrating advantages in patency, wound healing and the use for multiple revascularizations and sequential bypasses, the question of using a less traumatic device fulfilling the conditions of good coagulation, dissection of arterial branches, and dissection of perivascular tissue without damaging the arterial wall itself still remains [15, 16].

Over time, preparation of ITA grafts has evolved from the use of dissecting scissors and hemostatic clips, to conventional electrosurgery with continuous radiofrequency energy with different settings and output levels, to ultra-high radiofrequency “cold” knifes, and the use of ultrasonic devices [17–19]. Nevertheless, the established technique of using the conventional electrosurgical device at low output levels in many centers is still considered the standard procedure, which may be due to the generally low cost of the device, the non-superiority of other devices, further disadvantages of new devices and absent data concerning histological samples, long term outcomes results, or control groups [15–17, 20]. It still remains unknown what is the best method for ITA harvesting in a skeletonized fashion, treating the structural integrity of the artery as a risk factor of early and late graft failure.

Since 2008 the PEAK PlasmaBlade has been commercialized in Europe and the United States. Significant pre-clinical (animal) and clinical (human) research has been conducted with the device in complex settings, such as plastic and reconstructive, breast, and orthopedic surgery with good results concerning wound healing, damage of surrounding tissue, bleeding control and inflammation [21, 22]. To our knowledge, the PB has never been investigated concerning its effect on human vessel integrity. Our expectations regarding this new device in ITA harvesting were high, including a better integrity of the individual wall layers, and less damage of the endothelium secondary to lower heat transfer. As seen in other studies we expected an adequate bleeding control without adventitial hemorrhage around the vessel.

Concentrating on the intra- and immediate postoperative course, no outcome difference could be found between the test instruments in the study population. All of the patients were hemodynamically stable and received an off-pump bypass operation without any complications.

Histologically, comparison of all PB versus conventional electrosurgery samples demonstrated a significant benefit of the PB in reducing endothelial damage. The analysis also showed a tendency to better wall integrity, but this was not statistically significant. Concerning the adventitial bleeding, we found no difference between either group. Empirically, after vessel harvest with the PB the surrounding tissue bed required additional coagulation by the conventional electrosurgical device because of insufficient bleeding control with the PB. We interpret this result as the consequence of the active rim of the blade, which is the small area of heat development, and only demonstrates a limited surface for energy output, which might not be effective enough for hemostasis of a vessel stump. This led to a modification of the preparation technique in the sense that the distal part of the side branches had to be clipped as well.

Fourteen of the sixteen CTs demonstrated good patency of both ITAs. In the isolated case of the failed right ITA harvested with the PB, the right coronary artery target vessel had a stenosis of barely 50% with collateralization from the left descending artery. This was bypassed with the left ITA with a uniform patency. We interpret this occlusion more likely in the context of competitive flow from the left descending artery than being caused by the use of the PB itself. Moreover, the indication for the bypass to the right coronary artery in this case might be discussed. On another CT, both ITAs could not be localized. The single-photon emission computed tomography in this asymptomatic patient confirmed good viability of the myocardium in the target area. Further investigation via coronary angiography was not performed in this patient. We conclude from these results, that it might speak for a non-inferiority of the PB compared to conventional EC when considering six-month patency of the ITAs.

We also found a difference between both groups with regard to the time required for harvesting, which was on average 5 minutes longer in the PB group. The difference of preparation time can be answered by the coagulation function of the PB compared to the conventional electrosurgical device leading to a longer time for hemostatic control of the side branches, which could be performed safely without any relevant postoperative bleeding in all patients after the adjustment of the preparation technique as mentioned above.

Examining the costs for both single-use devices, the conventional electrosurgical device is substantially less expensive. The PB is priced competitively with comparable advanced electrosurgery systems, for example Ultrasonic Scalpels, ($200 to $300) in the United States. The comparison in our department resulted in 5.40 Euro for the conventional EC and 240 Euro for the PB, which does not allow for routine use of the PB at the moment.

Finally, in considering the atraumatic characteristics of the PB, the idea of it being a safer tool for training young surgeons to avoid local damage of the ITA during skeletonized preparation during their education should be considered.

### Limitations of the study

There are some limitations regarding the routine preparation of ITA harvesting. We compared our standard settings, which are mainly common for skeletonized harvesting, but might vary among other departments using different modes like “cut” and “coag” or different current output.

Moreover, we used the distal ends of the ITA, which leads to a selected range for the extraction area of the five samples that we investigated per ITA. Therefore we cannot give any evidence concerning the full length of the artery.

Finally, till now the histological results are not supported by functional tests, as this study demonstrates the first pilot investigation regarding the use on vessels. For evaluation of endothelial function further analysis of physiological integrity might be conceivable, which is going to be included in future investigations after these promising histological results.

## Conclusion

The histological data from this study demonstrate significant differences between the conventional electrosurgical device and the PlasmaBlade, demonstrating a more intact endothelium and a tendency to improved wall integrity, with no difference in adventitial bleeding. The graft patency of ITAs harvested with the PB, as determined by CT, was satisfactory with the exception of one case with a questionable bypass indication and might speak for non-inferiority to ITAs harvested by conventional electrosurgery. In expert hands the preparation time with the PB is few minutes longer. For training purposes during ITA harvesting, the PB could be considered as a safe and easily-handled device, possibly reducing risk from trainee error.

Further investigation, specifically in histological analysis, cardiac computed tomography of patency, and outcomes endpoints, is planned by future preparation with the PB and long time follow up.

## Abbreviations

Alcian blue-PAS: Alcian blue- periodic acid-Schiff
CD31: Cluster of differentiation 31
CT: Computed tomography
EC: Electrosurgical device
EvG: Elastica van gieson
H&E: Haematoxylin and eosin
ITA: Internal thoracic artery
PB: PlasmaBlade
